# Oxidative Stress and Erythrocyte Membrane Alterations in Children with Autism: Correlation with Clinical Features

**DOI:** 10.1371/journal.pone.0066418

**Published:** 2013-06-19

**Authors:** Alessandro Ghezzo, Paola Visconti, Provvidenza M. Abruzzo, Alessandra Bolotta, Carla Ferreri, Giuseppe Gobbi, Gemma Malisardi, Stefano Manfredini, Marina Marini, Laura Nanetti, Emanuela Pipitone, Francesca Raffaelli, Federica Resca, Arianna Vignini, Laura Mazzanti

**Affiliations:** 1 Department of Experimental, Diagnostic, and Specialty Medicine, University of Bologna, Bologna, Italy; 2 Neuropsichiatric Unit, Ospedale Maggiore, Bologna, Italy; 3 Don Carlo Gnocchi Foundation ONLUS, Milan, Italy; 4 ISOF, CNR, Bologna, Italy; 5 Department of Pharmaceutical Sciences, and AmbrosiaLab, University of Ferrara, Ferrara, Italy; 6 Dept of Clinical Sciences - Biochemistry, Polytechnic University of Marche, Ancona, Italy; 7 Azienda USL, Bologna, Italy; Torrey Pines Institute for Molecular Studies, United States of America

## Abstract

It has been suggested that oxidative stress may play a role in the pathogenesis of Autism Spectrum Disorders (ASD), but the literature reports somewhat contradictory results. To further investigate the issue, we evaluated a high number of peripheral oxidative stress parameters, and some related issues such as erythrocyte membrane functional features and lipid composition. Twenty-one autistic children (Au) aged 5 to 12 years, were gender and age-matched with 20 typically developing children (TD). Erythrocyte thiobarbituric acid reactive substances, urinary isoprostane and hexanoyl-lysine adduct levels were elevated in Au, thus confirming the occurrence of an imbalance of the redox status of Au, whilst other oxidative stress markers or associated parameters (urinary 8-oxo-dG, plasma radical absorbance capacity and carbonyl groups, erythrocyte superoxide dismutase and catalase activities) were unchanged. A very significant reduction of Na^+^/K^+^-ATPase activity (−66%, p<0.0001), a reduction of erythrocyte membrane fluidity and alteration in erythrocyte fatty acid membrane profile (increase in monounsaturated fatty acids, decrease in EPA and DHA-ω3 with a consequent increase in ω6/ω3 ratio) were found in Au compared to TD, without change in membrane sialic acid content. Some Au clinical features appear to be correlated with these findings; in particular, hyperactivity score appears to be related with some parameters of the lipidomic profile and membrane fluidity. Oxidative stress and erythrocyte membrane alterations may play a role in the pathogenesis of ASD and prompt the development of palliative therapeutic protocols. Moreover, the marked decrease in NKA could be potentially utilized as a peripheral biomarker of ASD.

## Introduction

Autism spectrum disorders (ASD) are complex neuro-developmental disorders characterized by impairment in social interaction and communication, and exhibition of repetitive and stereotypic behaviours. Diagnosis of ASD is based on clinical features only, and at present there are no validated biomarkers for diagnostic and/or screening purposes [1]. Genetic susceptibility, immunologic alterations, and environmental factors have been proposed to play an etio-pathogenic role in ASD [2]. It has been suggested that oxidative stress may play a role in the etio-pathogenesis of ASD [3]–[5]. Oxidative stress is defined as the disruption of the normal intracellular balance between reactive oxygen species (ROS), produced either during aerobic metabolism or as a consequence of pathologic processes and antioxidant defence mechanisms [6]. Oxidative stress, in turn, induces the secretion of numerous vasoactive and pro-inflammatory molecules [7] leading to neuroinflammation [2]. Oxidative stress has been suggested to underlie several other mental disorders, including schizophrenia and bipolar disorder [8]–[10], and neurodegenerative pathologies such as Alzheimer disease [11]. Oxidative stress is the result of increased production of pro-oxidant species or decreased antioxidant defences; glutathione redox status has indeed been found to be decreased in autistic patients, also in the post-mortem analysis of Autistic brain tissues [12].

Oxidative stress can be detected by studying a panel of different markers [13], some of which, such as DNA, proteins and polyunsaturated fatty acid (PUFA) residues, are pathognomonic of oxidative damage of biomolecules. It is worth mentioning that lipid peroxidation was found to be elevated in autism [14] and that PUFA are important for neurodevelopment [15]. Noteworthy, the imbalance of membrane fatty acid composition and PUFA loss can affect ion channels and receptors [16]. In particular, Ca^2+^ channel deficiency was found in Au [17], but never correlated to membrane parameters.

The aim of our study was to evaluate an integrated biomarker panel in Autistic (Au) children, in order to assess the possible imbalance of their redox status. The rationale for the choice of the parameters we examined was based on the strong correlation between: a) erythrocyte fatty acid membrane profile and preservation/degeneration of brain functions in aging and in neurodegenerative diseases [18], [19]; b) erythrocyte membrane ω6/ω3 balance and inflammation markers [20]; c) peripheral and central nervous system markers of oxidative stress [21]. All these biomarkers are components of an intertwined biological system, wherein erythrocyte membrane functional and structural characteristics act as a sensor of pathological changes. The recognition of biochemical alterations occurring in ASD subjects may also result in therapeutic methods aimed at reducing some of the symptoms. Also, the examined parameters are a potentially useful biomarker of ASD.

## Materials and Methods

### Ethics Statement

The present study was conducted according to the guidelines laid down in the Declaration of Helsinki and all procedures involving human patients were approved by Local Ethical Committee (Azienda USL Bologna, CE 10020- n.30, 06/04/2010 prot 45424/10-03). Written consent was obtained from all parents and also from children through pictures and simplified information.

### Subjects

A total of 48 children were approached as part of the present case–control study. Of these, 25 had a diagnosis of Autism (Au) and 23 were classified as Typically Developing (TD) children. Of these, 21 were recruited for inclusion in the study from the autism group (4 F and 17 M), and 20 in the TD group (6 F and 14 M). Reasons for rejection included: taking fish oil supplements (two subjects in the Au group), taking vitamins and/or other substance known to have antioxidant properties (two subjects in Au group and 3 subjects in TD group).

Au group mean age was 6.8 years (SD = 2.23 years, median = 6 years, range 5–12 years); in TD group mean age was 7.6 years (SD = 1.96 years, median = 7 years, range 5–12 years). Both the non-parametric comparison of the average age in the two groups and the comparison by gender (chi-square test), were not significant, confirming the comparability between cases and controls.

All the patients were admitted to Child Neuropsychiatric Unit of the Maggiore Hospital of Bologna (Neurological Sciences Institute IRCCS-Bologna), for assessment by a comprehensive diagnostic- neurological workup and regular follow-ups. None of the autistic patients had active epilepsy at the time of blood and urine sampling. One patient experienced a first and (at the moment of writing this paper) single benign rolandic seizure six months after the blood and urine collection (this patient was the only one with a normal intellectual level). Any medical and neurological comorbidity was excluded by electroencephalography (recorded during awake and sleep), cerebral magnetic resonance imaging, standard clinical and neurological examination, neurometabolic and genetic investigations (including 550 band karyotype, and molecular assay for Fragile X and MECP2). No infective or inflammatory disease was detected at the time of blood collection. No subject underwent any surgery intervention in the four months prior to blood and urine collection.

Autism diagnosis was made according to the Diagnostic and Statistical Manual of Mental Disorders IV (DSM IV TR [22]) criteria, Autism Diagnostic Observation Schedule (ADOS) [23] and Childhood Autism Rating Scale (CARS) [24] by two clinicians (a child neuro-psychiatrist and a child psychologist) experienced in the field of autism (P.V., F.R.). Developmental and cognitive levels were assessed by Psychoeducational Profile-3 (PEP-3) [25] and Leiter International Performance Scale–Revised (Leiter-R) [26]. Parents were questioned regarding the age of onset of early autistic signs. Demographic and clinical features of Au group are summarized in Table 1. Control group children were healthy TD children, recruited in the local community, with no sign of cognitive, learning and psychiatric involvement, as clinically and anamnestically determined by three experienced clinicians (A.G., P.V., F.R.). All TD were attending mainstream school and had not been subjected to stressful events. Dietary habits were assessed by a Food Questionnaire. All patients and controls were on a typical Mediterranean diet.

**Table 1:** Demographic and clinical features of the autistic children group.

No.	Gender	Age (months)	Age of onset (months)	Cognitive/developmental impairment	DSM IV-TRdiagnosis	ADOSscore	ADOSdiagnosis	CARSglobalscore	CARS activity level item score (hyperactivity)	CARS body use item score (stereotypes)
1	M	66	≤12	Moderate	PDD	18	Au	40.5	2.5	3.5
2	M	74	≤12	Severe	PDD	21	Au	42	2.5	2.5
3	M	64	≤12	Moderate	PDD	16	Au	35	2.5	2
4	M	103	13–18	Moderate	PDD	19	Au	44.5	3	3
5	M	61	≤12	Severe	PDD	21	Au	46	3.5	4
6	M	71	13–18	Severe	PDD	22	Au	41	2	3
7	M	142	≤12	Severe	PDD	22	Au	44.5	2.5	3
8	M	131	13–18	Severe	PDD-NOS	16	Au	38	3	3
9	M	66	13–18	Moderate	PDD	22	Au	40.5	3	3
10	F	66	13–18	Borderline IQ	PDD-NOS	15	Au	41.5	2.5	2
11	M	74	13–18	Severe	PDD	22	Au	42.5	3	2.5
12	F	66	13–18	Severe	PDD	22	Au	43.5	3.5	3.5
13	M	66	13–18	Mild	PDD-NOS	14	Au	34	2	2
14	M	89	19–24	Mild	PDD	19	Au	40	3	3
15	M	102	13–18	Mild	PDD	22	Au	36.5	2	2.5
16	F	110	25–30	Moderate	PDD	15	Au	47.5	3.5	3
17	M	79	13–18	Moderate	PDD	19	Au	37	2.5	3
18	M	144	13–18	Severe	PDD-NOS	20	Au	39	3	3.5
19	M	80	≤12	Normal IQ	PDD	19	Au	36.5	2.5	2
20	M	79	≤12	Severe	PDD	21	Au	40.5	2.5	3
21	F	65	≤12	Mild	PDD-NOS	17	Au	31.5	2	2

PDD: Pervasive Developmental Disorder; PDD-NOS: Pervasive Developmental Disorder-Not Otherwise Specified; Au: Autism.

### Biochemical Evaluations

Blood samples, obtained from Au and TD children, were collected in Na_2_-EDTA (∼9 mL) and heparin (∼5 mL) vacutainers. Some hematological parameters were carried out by routine laboratory techniques. One ml Na_2_-EDTA whole blood was set apart for lipidomics evaluation. The remaining blood was centrifuged (10 min. at 1000×g) in order to separate the plasma, which was frozen at −80°C in 1 mL eppendorf sterile tubes. Na_2_-EDTA and heparinised plasma was used for a radical absorbance capacity (ORAC) test and protein carbonyl evaluation, respectively. After diluting (1∶1) the cell suspension with sterile Phosphate Buffered Saline (PBS), mononuclear white blood cells were separated from red cells by Ficoll (Histopaque 1077, Sigma, St.Louis, MO, USA) density gradient centrifugation. Cells were lysed in 1 mL Trizol® Reagent (Invitrogen, Milan, Italy) and stored at −80°C for other evaluations. In order to remove all Ficoll residue red blood cells were washed three times with PBS. Erythrocytes in Na_2_-EDTA were stored at 4°C and then used for the evaluation of Na^+^/K^+^-ATPase activity (NKA) and cell membrane fluidity. Heparinised red blood cells (RBC) were used for the evaluation of superoxide dismutase (SOD) and catalase activity. In particular, for SOD activity measurement, heparinised RBC were lysed in 4 volumes of ice-cold water and then stored at −80°C. The remaining heparinised RBC were diluted 30-fold in PBS and subsequently lysed in 10 mM potassium phosphate buffer pH 7.2. Lysates were stored at −80°C and subsequently were used for catalase activity evaluation. Spot urine samples (10 mL) from Au and TD were collected. Proteinuria and creatinine determinations were carried out by laboratory techniques. The remaining urine was centrifuged at 1200 g for 10 min in order to remove insoluble materials. Five mL of clear urine were aliquoted and stored at −80°C for hexanoyl-lysine adduct (HEL) and 8-isoprostane evaluations. The remaining urine was filtered with 0,45 µm filter, supplemented with 0.05% sodium azide and stored at −80°C for 8-hydroxy-2′-deoxyguanosine (8-oxo-dG) analysis.

#### Urinary 8-isoprostane

Urinary 8-isoprostane (also known as 8-epi-PGF_2α_, 8-iso-PGF_2α_ or 15-isoprostane F_2t_) was determined by the use of a competitive ELISA kit (Oxford Biomedical Research Inc., Oxford, MI, USA). As suggested by the manufacturer, urine samples are diluted 1∶5 with a buffer provided in the kit. The 15- isoprostane F_2t_ in the samples competes with 15-isoprostane F_2t_ conjugated to horseradish peroxidase (HRP) for binding to a polyclonal antibody specific for 15-isoprostane F_2t_ coated on the microplate. A substrate was added and the absorbance was measured at 450 nm in a microplate reader. The 15-isoprostane F_2t_ concentration was expressed in ng per milligram of creatinine.

#### Urinary hexanoyl-lysine adduct

Hexanoyl-lysine adduct (HEL) concentration was measured by a competitive ELISA kit (JaICA, Fukuroi, Shizuoka, Japan) in unfiltered urine of autistic and control children. According to the manufacturer's instructions, urine samples were diluted five times with PBS. Some urine samples containing proteins were treated with 14 mg/mL alpha-chymotrypsin in PBS (pH 7.4) and incubated at 37°C O.N. Samples were filtered using ultra filters with cut-off molecular weight 10 kDa (Amicon Ultra, Millipore, Cork, Ireland). The absorbance was measured at 450 nm using a microplate reader. The HEL concentration was expressed in nmol per milligram of creatinine (nmol/mg creatinine).

#### Urinary 8-oxo-dG

Urinary 8-hydroxy-2′-deoxyguanosine (8-oxo-dG) was measured using the HT 8-oxo-dG ELISA Kit (Trevigen Inc. Gaithersburg, MD, USA) according to the manufacturer’s instructions. Briefly, filtered urine was diluted 1∶20 with a buffer provided by the kit and added to a plate pre-bounded with 8-oxo-dG. Bound and sample 8-oxo-dG compete for binding to the anti-8-oxo-dG which was then added to the plate; the antibody fraction captured by the immobilized 8-oxo-dG in the plate was then detected by means of a HRP-conjugated secondary antibody. The assay was developed with tetramethylbenzidine substrate (TMB) and the absorbance was measured in a microplate reader at 450 nm. The 8-oxo-dG concentration was expressed in ng per milligram of creatinine.

#### Protein carbonyl determination

Protein carbonyls were determined in plasma samples using the Protein Carbonyl ELISA kit (Enzo Life Sciences Inc. Farmingdale, NY, USA) following the manufacturer’s instructions. Plasma (5 µL) was derivatized with dinitrophenylhyidrazine (DNPH); derivatized proteins were then adsorbed to an ELISA plate. The adsorbed protein was then probed with biotinylated anti-DNP antibody followed by streptavidin-linked horseradish peroxidase. The absorbance was read at 450 nm using a spectrophotometer plate reader (Victor II, Pelkin-Elmer, Waltham, MA, USA). Plasma samples were assayed in duplicate, and protein carbonyl concentration was expressed as nanomoles of carbonyl groups per milligram of protein in the sample (nmol/mg).

#### Plasma radical absorbance capacity (ORAC)

The ORAC assay was carried out on a Fluoroskan FL® ascent (Thermo Fisher Scientific, Inc. Waltham, MA, USA) with fluorescent filters (excitation wavelength: 485 nm; emission filter: 538 nm). following a previously published procedure [27].

Briefly, in the final assay mixture (0.2 mL total volume), fluorescein sodium salt (85 nM) was used as a target of free radical attack with 2,2′-azobis(2-amidino-propane) dihydrochloride (AAPH) as a peroxyl radical generator. Trolox, a water-soluble analogue of vitamin E, was used as a standard control and calibration curves were determined for 10, 20, 30, 40, 50 µM solution. Fluorescence measurements, carried out at 37°C, were recorded at 5 min intervals, up to 30 min after the addition of AAPH. The ORAC values, calculated as difference of the areas under the quenching curves of fluoresceine between the blank and the sample, were expressed as Trolox equivalents (TE), pH = 7.4. All the assays were performed with three replicates.

#### Superoxide dismutase (SOD) activity

SOD activity was determined in erythrocyte lysates by a competitive colorimetric inhibition assay, as previously described [28], [29]. This method is based on water-soluble tetrazolium salt, WST-1 (2-(4-Iodophenyl)-3-(4-nitrophenyl)-5-(2,4-disulfophenyl)-2H-tetrazolium, monosodium salt) (Dojindo Laboratories Co., Kumamoto, Japan), which produces a water-soluble formazan dye upon reduction with the superoxide anion generated by xanthine and xanthine oxidase (Sigma-Aldrich, St. Louis, MO, USA). SOD activity reduces the superoxide concentration and inhibits formazan formation. A SOD standard curve was obtained; different dilutions of erythrocyte lysates were assayed in order to find a sample dilution that falls within the range of standard curve linearity. Samples or standards (10 µL) were incubated for 20 min at 37°C with 100 µL reaction mixture containing 500 µM WST-1 and 75 µM xanthine in 50 mM CHES (2-N-(Cyclohexylamino) ethanesulphonic acid, pH 8.0. Finally, 10 µL Xanthine Oxidase (350 mU/mL) (Sigma-Aldrich, St. Louis, MO, USA) was added. Formazan formation was measured at 450 nm using a 96-well plate reader (Victor2 Multilabel Counter, Perkin-Elmer, Waltham, MA, USA). SOD concentration, expressed in units per milligram of hemoglobin, was determined using the SOD standard curve.

#### Catalase activity

Catalase activity was determined in erythrocyte lysates using a method described by Ou and Wolff [30], based on the specific reaction of FOX-1 reagent (250 µM ammonium ferrous sulfate, 100 µM xylenol orange, 0,1 M sorbitol, 25 mM H_2_SO_4_) with H_2_O_2_ to yield a color complex having absorption maximum at 560 nm. The catalase causes decomposition of H_2_O_2_ such that residual H_2_O_2_ is inversely proportional to the activity of the catalase. One milliliter of erythrocyte lysates was incubated for 4 min. with 100 µL of 2.2 mM H_2_O_2_. Subsequently, 50 µL aliquots of the incubation mixtures were removed and rapidly mixed with 950 µL of FOX-1 reagent in eppendorf tubes, which were then incubated at room temperature for 30 min. Absorbance was measured at 560 nm. Catalase concentration was expressed in units per milligram of hemoglobin.

#### Erythrocyte plasma membrane fluidity

Erythrocytes plasma membrane fluidity was studied by determining the fluorescence anisotropy (reciprocal of fluidity) of two probes, TMA-DPH (1-(4-trimethylammoniophenyl)-6-phenyl-1,3,5-hexatriene), and DPH (1-6-phenyl-1,3,5-hexatriene); used to evaluate membrane fluidity of the outer and the inner leaflet of cell membrane, respectively [31]. The fluorescent probes were purchased from Molecular Probes Inc (Eugene, OR, USA). The incubation with TMA-DPH and DPH was performed as described by Sheridan and Block [32]. Briefly, 3 µl of TMA-DPH and DPH (10^−3^ mol/L) were incubated for 5 min and 45 min respectively, at room temperature (23°C) with 2 ml of erythrocyte membranes (final concentration of 100 µg/mL) in 50 mmol/L Tris-HCl buffer solution, pH 7.4. Fluorescence intensities (100 readings each) of the vertical and horizontal components of the emitted light were measured on a Perkin-Elmer MPF-66 spectrofluorometer equipped with two glass prism polarizers (excitation wavelength 365 nm, emission wavelength 430 nm). Sample temperature was maintained at 37°C using an external bath circulator (Haake F3). Steady-state fluorescence anisotropy (r) of TMA-DPH and DPH was calculated using the equation.
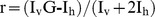
where G is an instrument factor correcting for unequal detection of vertically (I_v_) and horizontally (I_h_) polarized light.

#### Na^+^/K^+^-ATPase activity

Na^+^/K^+^-activated Mg^2+^-dependent ATPase activity was determined in cell membranes by the Kitao method [33]. ATPase activity was assayed by incubating 1 mL of erythrocyte plasma membrane after sonication (three bursts, 15 s each) at 37°C in a reaction medium containing MgCl_2_ (5 mmol/L), NaCl (140 mmol/L), KCl (14 mmol/L) in 40 mmol/L Tris-HCl, pH 7.7. The ATPase reaction was initiated with the addition of 3 mmol/L Na_2_ATP and stopped 20 min later by the addition of 1 mL of 15% trichloracetic acid. The tubes were then centrifuged at 1100 g for 10 min and the inorganic phosphate (P_i_) hydrolysed from the reaction was measured in the supernatant by a colourimetric assay using a KH_2_PO_4_ standard [34]. ATPase activity, assayed in the presence of 10 mmol/L ouabain, was subtracted from the total Mg^2+^-dependent ATPase activity to calculate the activity of Na^+^/K^+^-ATPase. Protein concentration was determined as described by Bradford [35], using serum albumin as a standard. The interassay variation was 5.3%, while the intra-assay variation was 8.1%.

#### Lipoperoxide levels (TBARs) measurement

Lipoperoxide levels were evaluated using Cayman's thiobarbituric acid reactive substances (TBARs) assay kit. The product of fatty acid peroxidation, malondialdehyde (MDA), reacts with thiobarbituric acid (TBA) to yield a product that is measured fluorometrically. Membranes (100 mg of membrane proteins) were centrifuged at 3000 g for 15 min after the addition of 30% trichloroacetic acid, and 0.5 mL of the resulting supernatant was mixed with 1.1 mL of TBA reagent (equal volumes of 0.67% TBA aqueous solution and glacial acetic acid; v/v). The reaction mixture was heated for 60 min at 95°C in a sand bath. After cooling to room temperature, 5 mL of n-butanol was added and the mixture was shaken vigorously for 2 min. Thereafter, samples were centrifuged at 4000 g for 15 min, then 150 µL from each vial were loaded to the plate for fluorometric assay and the fluorescence of samples and standards was read at an excitation wavelength of 530 nm and an emission wavelength of 550 nm. The lipid peroxide level (Lp) was expressed in terms of MDA content (µM), using 1,1′,3,3′-tetramethoxypropane as a standard.

#### Sialic acid

Sialic acid content of RBC membranes was determined by the periodate thiobarbituric acid method of Denny et al. [36]. Briefly, membranes (1 mg membrane proteins/mL) were first hydrolyzed in 0.05-mol/L H_2_SO_4_ in a final volume of 0.1 mL for 1 hour at 80°C to release SA [37]. Standards and samples were both incubated with (assay samples) or without (correction samples) 0.25 mL periodate solution (0.025 mol/L periodic acid in 0.25 mol/L HCI) at 37°C for 30 minutes [38]. After reduction of excess periodate with 0.25 mL 0.32 mol/L sodium thiosulfate, the reaction was completed by addition of 1.25 mL 0.1-mol/L thiobarbituric acid. The samples were heated at 100°C for 15 minutes and then cooled to room temperature. The product was extracted with acidic butanol and colorimetrically assayed with a spectrophotometer at 549 nm. The readings of correction samples were subtracted from those of assay samples, thus corrected readings were obtained.

Protein content was determined by Bradford method to normalize the sialic acid content using BSA as standard [35].

#### Erythrocyte membrane lipidomic analysis

The erythrocyte fatty acid membrane profile analysis was carried out as previously described, using the erythrocyte membrane pellet obtained by standard methods [39]. For this study, selection of the erythrocyte fraction was made by modification of a literature procedure for the selection of aged erythrocytes (red blood cell age >3 months), with cells selected for high density and small diameter compared to the average erythrocyte population [40].

One mL of whole blood was first centrifuged at 2000 g for 5 min to eliminate the plasma, and a second round of centrifugation was then carried out at 4000 g at 4°C for 5 min in order to yield a stratification by cell density. The bottom layer (2.5 mm from the bottom of tube) consisted of erythrocyte cells, which were evaluated for their diameter using a Scepter™ 2.0 Cell Counter (Merck Millipore, Milan, Italy) to characterize the cell selection from each blood sample. The results were also compared with the cell population obtained from standard density gradient separation [41], [42].

Briefly, lipids were extracted from erythrocyte membranes according to the method of Bligh and Dyer [43]. The phospholipid fraction was controlled by TLC as previously described [39], then treated with KOH/MeOH solution (0.5 M) for 10 min at room temperature and under stirring [44].

Fatty acid methyl esters (FAME) were extracted using n-hexane; the hexane phase was collected and dried with anhydrous Na_2_SO_4_. After filtration, the solvent was eliminated by evaporation using a rotating evaporator, and the thin white film of the FAME was subsequently dissolved in a small volume of n-hexane. Approximately 1 µL of this solution was injected into the GC. A Varian CP-3800 gas chromatograph, with a flame ionization detector and an Rtx-2330 column (90% biscyanopropyl-10% phenylcyanopropyl polysiloxane capillary column; 60 m, 0.25 mm i.d., 0.20 µm film thickness) was used for the analysis. Temperature was held at 165°C held for the initial 3 min, followed by an increase of 1°C/min up to 195°C, held for 40 min, followed by a second increase of 10°C/min up to 250°C, held for 5 min. The carrier gas was helium, held at a constant pressure of 29 psi. Methyl esters were identified by comparison with the retention times of commercially available standards or trans fatty acid references, obtained as described elsewhere [45].

### Statistics

All experiments were carried out in duplicate or triplicate and were usually repeated three times.

To compare Au and TD groups, normality tests were applied to all numeric variables, following which appropriate parametric tests (ANOVA, Student's t for independent data) or the nonparametric equivalent (Wilcoxon-Mann-Whitney) were used. Non-parametric correlation (Spearman's rho) was used to correlate clinical features and biochemical data in the Au group (non-parametric ANOVA for cognitive/developmental level). Differences were considered significant at p<0.05.

To account for multiple testing we used the Benjamini and Hochberg false discovery rate (FDR) [46]. FDR corrected p-values (*pFDR*) were evaluated separately for a) comparisons of biochemical parameters in Au and TD and b) correlations of clinical features and biochemical data in Au. In particular, the comparisons of biochemical parameters included a1) erythrocyte membrane features and molecules, oxidative stress markers (in urine and plasma) and antioxidant enzyme activities in erythrocytes (12 comparisons); a2) erythrocyte membrane fatty acids (19 comparisons). As for the correlations between Au clinical features and biochemical data, *pFDR* was calculated for CARS global score (31 comparisons), CARS activity level (hyperactivity) item (31 comparisons), CARS body use (stereotypes) item (31 comparisons), cognitive/developmental impairment levels (31 comparisons). Age was compared with all biochemical data (31 comparisons).

Even though it is usual to set at <0.05 the significance level of statistic tests, Benjamini & Hochberg [46], as well as others [47], have argued that a more liberal threshold (as high as 0.1 or even a bit higher) may be reasonable for *pFDR*.

Statistical analysis was performed using SAS v. 9.2.

## Results

### 1. Comparisons between Au and TD

#### 1.1 Oxidative stress markers in urine and plasma and antioxidant enzymes activities in erythrocytes (Fig. 1 and table 2)

Peroxidation of arachidonic acid causes membranes to release 8-isoprostane, a prostaglandin-F2-like compound. Oxidized arachidonic acid or other omega-6 fatty acids, such as linoleic acid, may also react with protein lysine residues, yielding HEL. Both 8-isoprostane (p<0.01; *pFDR = *0.0278) and HEL (p<0.05; *pFDR = *0.076) were found in higher amount in the urine of Au than in the urine of TD children (+47% and +45%, respectively). However, the amount of 8-oxo-dG, derived from the oxidation of nucleic acid bases by free radicals, did not significantly differ between the two groups.

**Figure 1:**
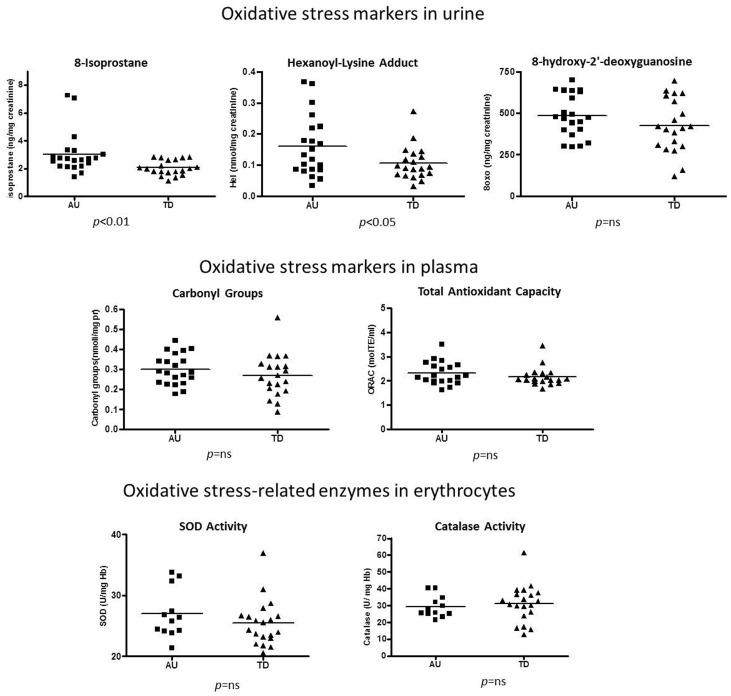
Scatter plot showing oxidative stress markers in urine and plasma and antioxidant enzymes activities in erythrocytes. Au = Autistic children; TD = typically developing children. Horizontal bars indicate means. Standard deviation values and whether parametric or not parametric statistic tests were applied, are reported in Tab. 2. p<0.01 highly significant; p<0.05 significant; *ns*, not significant.

**Table 2:** Erythrocyte membrane features and molecules, oxidative stress markers in urine and plasma, antioxidant enzymes activities in erythrocytes.

	Mean values ± St. Dev		% difference Au vsTD	Statistical significance		
	Au (N = 21)	TD (N = 20)		p values		*pFDR*
**Erythrocyte membrane features and molecules**						
Na^+^/K^+^-ATPase°	2.54±0.58	7.39±1.62	**−66%**	**<0.0001**	<0.0001	
TMA-DPH°	0.27±0.02	0.25±0.03	**+8%**	**0.0123**	0.0368	
DPH°	0.27±0.02	0.25±0.03	**+8%**	**0.0196**	0.0469	
TBARS°	0.72±0.38	0.51±0.37	**+41%**	**0.0021**	0.0125	
Sialic Acid°	6.19±4.36	7.63±7.08	−19%	0.7248	0.7248	
**Oxidative stress markers in urine**						
8-Isoprostane°	3.04±1.50	2.07±0.54	**+47%**	**0.0069**	0.0278	
HEL*	0.16±0.09	0.11±0.05	**+45%**	**0.0380**	0.0760	
8-Oxo-dG*	484.80±130.07	426.46±163.64	+14%	0.2127	0.346	
**Oxidative stress markers in plasma**						
Carbonyl Groups*	0.30±0.08	0.27±0.11	+11%	0.2509	0.3763	
ORAC°	2.47±0.86	2.36±0.94	+5%	0.4573	0.5487	
**Antioxidant enzymes activities in erythrocytes**						
SOD activity° (Au N = 12)	26.10±4.02	25.49±3.78	+2%	0.2960	0.3947	
Catalase activity* (Au N = 12)	29.28±6.34	31.25±11.06	−6%	0.5783	0.6309	

Au: Autistic children; TD: typically developing children; p values were calculated with non parametric Wilcoxon-Mann-Whitney test (°) or parametric ANOVA test (*); **p<0.05**: significant; **p<0.01** highly significant; *pFDR*: Benjamini and Hochberg False Discovery Rate (FDR) corrected p-values.

Plasma levels of carbonyl groups (an oxidative modification of proteins) and plasma radical absorbance capacity (a measure of the antioxidant capacity, which is reduced by free radicals) did not differ between the two groups.

Similarly, neither SOD nor catalase enzymatic activity measured in erythrocytes were found to differ between the two groups.

#### 1.2 Erythrocyte membrane features and molecules (Fig. 2 and table 2)

TMA-DPH and DPH are two probes used to evaluate membrane fluidity of the outer and the inner leaflet of cell membrane, respectively. Taking into account that TMA-DPH and DPH fluorescence anisotropy is inversely related to the fluidity of the microenvironment where the probe is located, it was found that membrane fluidity was decreased in Au with respect to TD. The decrease reached the statistical significance (p<0.05) for both the outer and inner membrane (*pFDR = *0.0368, *pFDR = *0.0469, respectively).

**Figure 2:**
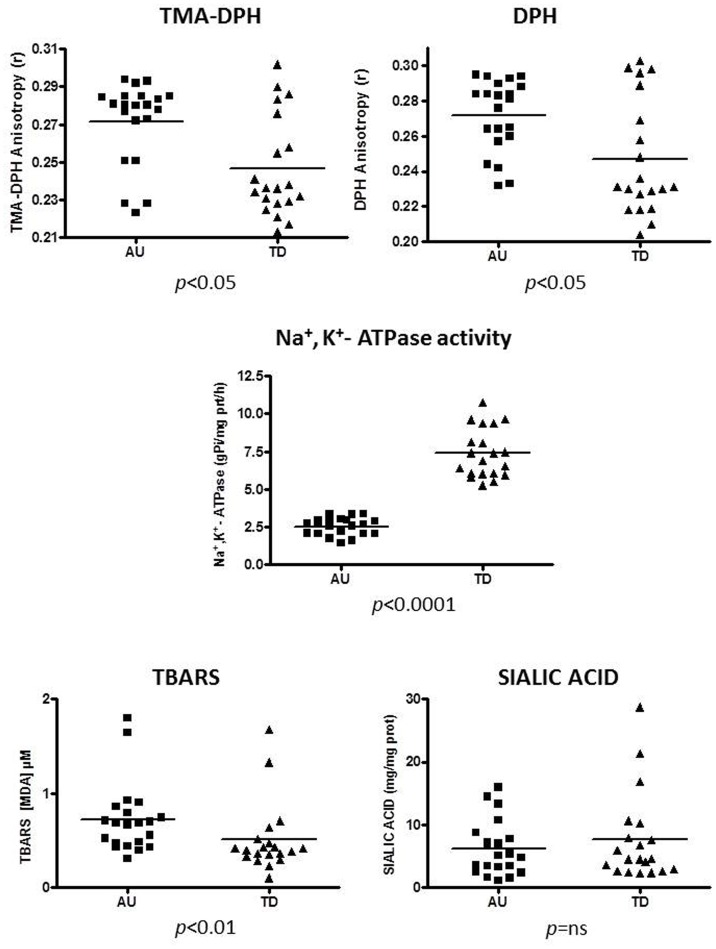
Scatter plot showing erythrocyte membrane features and molecules. Au = Autistic children; TD = typically developing children. TMA-DPH and DPH values are inversely correlated with the outer and the inner membrane fluidity, respectively. TBARS = Thiobarbituric Acid Reactive Substances. Horizontal bars indicate means. Standard deviation values and whether parametric or not parametric statistic tests were applied, are reported in Tab. 2. p<0.01 highly significant; p<0.05 significant *ns*, not significant.

The activity of Na^+^/K^+^-ATPase, an active ion transporter localized in the plasma membrane, was markedly decreased (−66%) in Au in comparison with TD (p<0.0001; *pFDR*<0.0001), with no overlapping values between Au and TD (Au min. 1.41, max. 3.38; TD min. 5.27, max. 10.75).

TBARS assay measures MDA generated from the decomposition of primary and secondary lipid peroxidation products. TBARS were found to be significantly higher (+41%) in the erythrocyte membrane from Au children in comparison with those from TD children (p<0.01; *pFDR* = 0.0125).

Sialic acid levels in erythrocyte did not differ between Au and TD.

#### 1.3 Analysis of erythrocyte membrane fatty acids (Table 3)

The percentage of oleic, palmitoleic and vaccenic acids and, in general, total MUFA were increased in Au with respect to TD children. This caused also a decrease in SFA/MUFA ratio in Au with respect to TD children (p<0.05; *pFDR* = 0.07329).

**Table 3:** Erythrocyte membrane Fatty Acid profile.

	Mean values (± St. Dev)		% differencesAu vs TD	Statistical significance	
	Au (N = 21)	TD (N = 20)		p values	*pFDR*
DHA omega 3 (22∶6)*	4.80±1.08	5.62±0.67	**−14%**	**0.0065**	0.07220
Total Monounsaturated Fatty Acids (MUFA)*	18.03±1.25	17.04±0.98	**+6%**	**0.0076**	0.07220
**Vaccenic acid (18∶1)°**	1.30±0.16	1.20±0.11	**+9%**	**0.0220**	0.07329
Oleic acid (18∶1)*	16.42±1.25	15.60±0.94	**+5%**	**0.0228**	0.07329
**SFA/MUFA°**	2.38±0.24	2.52±0.19	**−5%**	**0.0232**	0.07329
**Palmitoleic acid (16∶1)°**	0.3±0.08	0.24±0.09	**+25%**	**0.0262**	0.07329
ω6/ω3 ratio*	6.66±1.62	5.76±0.67	**+16%**	**0.0270**	0.07329
**EPA omega 3 (20∶5)°**	0.43±0.16	0.51±0.14	**−16%**	**0.0434**	0.10308
Total Polyunsaturated Fatty Acids (PUFA)°	39.40±1.80	40.18±1.74	−2%	0.1173	0.24763
Trans 18∶1°	0.11±0.05	0.14±0.07	−21%	0.1863	0.35397
Eicosatrienoic acid omega 6 (20∶3)°	2.25±0.45	2.13±0.34	+6%	0.3714	0.64151
Stearic acid (18∶0)*	18.58±1.04	18.78±0.88	−1%	0.4940	0.76378
Total Saturated Fatty Acids (SFA)*	42.31±1.96	42.60±1.53	−1%	0.6012	0.76738
Linoleic omega 6 (18∶2)°	12.2±0.96	12.66±1.34	−4%	0.6012	0.76738
Total TRANS°	0.23±0.08	0.24±0.06	−1%	0.6180	0.76738
Trans-ARA°	0.12±0.06	0.11±0.03	+9%	0.6756	0.76738
Arachidonic acid omega 6 (20∶4)*	19.57±1.67	19.39±1.13	+1%	0.6866	0.76738
EFA deficiency°	0.66±0.07	0.67±0.08	−1%	0.7961	0.84033
Palmitic acid (16∶0) *	23.73±1.94	23.82±1.48	0%	0.8810	0.88100

Au: Autistic children; TD: typically developing children; ARA, arachidonic acid; DHA, docosahexaenoic acid; EFA, essential fatty acids; EPA, eicosapentaenoic acid; MUFA, monounsaturated fatty acids; PUFA; polyunsaturated fatty acids; SFA, saturated fatty acids; TRANS, transfatty acids; p values were calculated with non parametric Wilcoxon-Mann-Whitney test (°) or parametric ANOVA test (*); **p<0.05**: significant; **p<0.01** highly significant; *pFDR*: Benjamini and Hochberg False Discovery Rate (FDR) corrected p-values.

The relative amount of the different PUFA was also altered, since EPA and DHA-ω3 acids were decreased in Au children (−16%, p<0.05, *pFDR* = 0.10308 and −14%, p<0.01, *pFDR* = 0.0722, respectively), causing an increase in ω6/ω3 ratio (+16%, p<0.05, *pFDR* = 0.07329). The results were interpreted using the fatty acid-based functional lipidomic approach [48].

### 2. Correlation between Au Clinical Features and Biochemical Data (Main Results Reported in Fig. 3 and Table 4)

Non-parametric correlation (Spearman's rho) was used to correlate clinical features and biochemical data in the Au group. CARS global scores were inversely related with ω6 arachidonic acid (p<0.05; *pFDR = *0.31104) and PUFA (p<0.05; *pFDR* = 0.18450). CARS activity level item scores (hyperactivity) were negatively correlated with TMA-DPH (p<0.01; *pFDR* = 0.03720), oleic acid (p<0.05; *pFDR* = 0.15035), ω6 arachidonic acid (p<0.05; *pFDR* = 0.15035), MUFA (p<0.05; *pFDR* = 0.11728) and PUFA (p<0.01; *pFDR* = 0.03720), and directly correlated with SFA (p<0.001; *pFDR* = 0.00930), palmitic acid (p<0.01; *pFDR* = 0.03720), SFA/MUFA (p<0.001 *pFDR* = 0.03720). TMA-DPH was correlated with age (p<0.01 *pFDR* = 0.2376). CARS body use item scores (stereotypes) were not significantly related to any biochemical marker.

**Figure 3:**
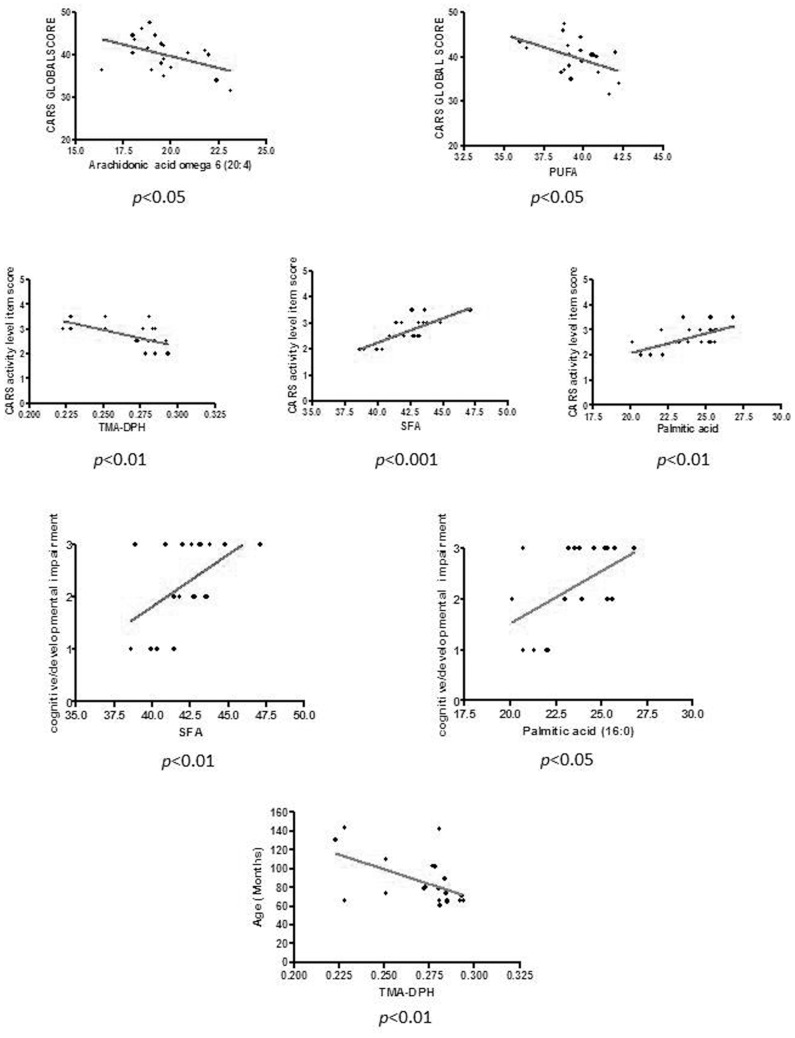
Relevant correlations between Au clinical features and biochemical data. Au patients were divided into three levels of cognitive/developmental impairment as follows: 1: mild, 2: moderate, 3: severe. TMA-DPH values are inversely correlated with the outer membrane fluidity. SFA = Saturated Fatty Acids. CARS activity level item score denotes hyperactivity. p<0.01 highly significant; p<0.05 significant. More details are reported in Tab.4.

**Table 4:** Significant correlations of clinical features and biochemical data in Autistic children.

	CARS global scores	CARS activity item (Hyperactivity)	Cognitive/developmental impairment level	Age
Total SFA	NS	**r = 0.70834; p<0.001;** *pFDR = *0.00930	**p<0.05;** *pFDR = *0.33199	NS
SFA/MUFA	NS	**r = 0.57825; p<0.001;** ***pFDR = *** **0.03720**	NS	NS
TMA-DPH	NS	**r** *** = −*** **0.58923; p<0.01;** *pFDR = *0.03720	NS	**r −0.6054; p<0.01;** *pFDR = *0.2376
Total PUFA	**r = −0.52589; p<0.05;** *pFDR = *0.18450	**r = −0.58719; p<0.01;** *pFDR = *0.03720	p = 0.0553; *pFDR = *0.33199	NS
Palmitic acid (16∶0)	NS	**r = 0.59763; p<0.01;** *pFDR = *0.03720	**p<0.05;** *pFDR = *0.33199	NS
Arachidonic acidω6 (20∶4)	**r = −0.432: p<0.05;** *pFDR = *0.31104	**r = −0.45377; p<0.05;** *pFDR = *0.15035	**p<0.05;** *pFDR = *0.33199	NS
Total MUFA	NS	**r = −0.49446; p<0.05;** *pFDR = *0.11728	NS	NS
Oleic acid (18∶1)	NS	**r = −0.46048; p<0.05;** *pFDR = *0.15035	NS	NS
8-isoprostane	NS	NS	**p<0.05;** *p*FDR = 0.33199	NS

Non-parametric correlation (Spearman's rho, r) was used to correlate clinical features (CARS, CARS activity item and Age) and biochemical data in the Autistic children group. Non-parametric ANOVA was used for Cognitive/developmental impairment level. MUFA, monounsaturated fatty acids; PUFA; polyunsaturated fatty acids; SFA, saturated fatty acids. **p<0.05**: significant; **p<0.01** highly significant.

*pFDR*: Benjamini and Hochberg False Discovery Rate (FDR) corrected p-values.

When only cognitive/developmental impaired Au children (n: 19) were considered, the non parametric ANOVA revealed that the level of cognitive/developmental impairment was inversely related with ω6 arachidonic acid (p<0.05; *pFDR* = 0.33199), and directly related with 8-isoprostane (p<0.05; *pFDR* = 0.33199), total SFA (p<0.05; *pFDR* = 0.33199) and palmitic acid (p<0.05; *pFDR* = 0.33199), while cognitive impairment and total PUFA showed only a trend of inverse correlation (p = 0.0553; *pFDR* = 0.33199).

Even if it was beyond the scope of this research, additional correlations were performed within Au clinical features. We found a significant correlation between CARS global score and other clinical features, such as cognitive/developmental delay (r = 0.52009, p<0.05; *pFDR = *0.18450), hyperactivity (r = 0.61669, p<0.01; *pFDR = *0.10440), CARS body use item scores (stereotypes) (r = 0.52009, p<0.01; *pFDR = *0.18450). Moreover, the variable stereotypes was related to CARS activity levels item score (hyperactivity) (r = 0,60308, p<0.01; *pFDR = *0.03060).

### 3. Statistics

FDR analysis confirmed the statistical significance of most uncorrected p values in both comparisons Au vs TD and in correlations between clinical features and biochemical parameters.

## Discussion

There is increasing evidence that autistic patients show excessive ROS production and several studies reported the presence of different peripheral biomarkers of oxidative stress [13], [49], [50]. However, the great heterogeneity of the syndrome makes it difficult to assess whether this finding is occasional and whether it is restricted to a sub-group of patients. Moreover, not all oxidative stress markers appear to be altered in patients, and it is still unknown whether oxidative stress, if really present, is secondary to a generic inflammatory status or due to genetic alterations still to be recognized. In addition, most researchers addressing this problem have a tendency to evaluate few markers at a time, thus making it very difficult to compare data obtained in different patient’s subgroups [51], [52]. To our best knowledge, this is the first study, which evaluated, at the same time, a wide range of different but strongly related biological biomarkers in a group of Au children that underwent a rigorous clinical characterization. Among the oxidative stress parameters we evaluated, we found, in Au compared to TD, a significant increase in TBARS, 8-isoprostane and HEL, which are markers of lipid peroxidation. No significant differences were found in the oxidative biomarkers 8-oxo-dG and ORAC. This finding suggests that the oxidative stress-related phenomena are localized mainly at the cell surface. Systemic involvement is suggested by detection of these markers both in urine and in erythrocytes.

The fatty acid composition of the brain and neural tissues is characterized by high PUFA concentrations, which play a very important role in signal transduction [53], neuro-inflammation [54] and cellular repair and survival [55]. Erythrocyte membrane fatty acid composition is a very sensitive indicator of the status of different tissues and may reflect the fatty acid composition of brain [56]. In a number of neurodevelopmental conditions, including Attention Deficit Hyperactivity Disorder (ADHD) and dyslexia, reduced concentrations of erythrocyte membrane PUFA have been reported [57]. Moreover, a polymorphism in the gene cluster associated with the fatty acid desaturase-2 gene (FADS2) for Delta 6-desaturase (the rate-limiting step in PUFA synthesis) was described in patients with ADHD [58], [59], pointing to a possible correlation between membrane fatty acid composition and hyperactivity.

Table 5 summarizes published data about alterations in erythrocyte membrane fatty acid composition in ASD children. In our study, a significant increase of erythrocyte membrane MUFA and of ω6/ω3 ratio (due to a decrease in EPA and DHA) was shown. These results are partially superimposable to those reported by Bell et al. [60]. Alteration in membrane lipid composition was not related to dietary habits, since they did not significantly differ between Au and TD, as evidenced by the Food Questionnaire. On the other hand, oxidative stress is not a likely explanation for the specific decrease of the ω3, since this would have also affected the ω6 PUFA family. The observed imbalance in ω6/ω3 ratio may lead to the proinflammatory status reported previously in ASD children [12], [61], [62]. The significant increase in MUFA may be representative of a feedback remodelling of erythrocyte membrane lipid composition. It is interesting to note that a study on adipocyte membranes showed DHA loss coexistent with MUFA increase [63].

**Table 5:** Summary of published results on fatty acid composition of erythrocyte membrane.

	fatty acid composition of erythrocyte membrane	Patients
Highly unsaturated fatty acids (HUFA)	decreased	One ASD patient [75]
Stearic acid (18∶0)	increased	18 Au children with developmental regression [76]
Arachidic acid (24∶0)	increased	
Total SFA	increased	
Oleic acid (18∶1 n-9)	decreased	
Nervonic acid (24∶1)	increased	
Total MUFA	decreased	
Linoleic acid (18∶2 n-6)	increased	
Arachidonic acid (ARA) (20∶4 n-6)	decreased	
Docosapentaenoic acid (DPA) (22∶5 n-6)	increased	
Docosapentaenoic acid (DPA) (22∶5 n-3)	decreased	
Total ω3	decreased	
ARA:EPA ratio (20∶4 n-6/20∶5n-3)	increased	
Stearic acid (18∶0)	increased	11 children with classical autism or Asperger [76]
Arachidic acid (24∶0)	increased	
Nervonic acid (24∶1)	increased	
Docosapentaenoic acid (DPA) (22∶5 n-6)	increased	
Docosapentaenoic acid (DPA) (22∶5 n-3)	decreased	
Total ω3	decreased	
ARA:EPA ratio (20∶4 n-6/20∶5n-3)	increased	
Eicosenoic acid (20∶1n9)	increased	20 Au children with developmental regression (mean age 3.5 years) [77]
Erucic acid (22∶1n9)	increased	
total MUFA	increased	
α-Linolenic acid (18∶3 n-3)	decreased	49 Au children (mean age 7.5 years) [78]
ARA:EPA ratio (20∶4 n-6/20∶5 n-3)	increased	

ARA, arachidonic acid; EPA, eicosapentaenoic acid; MUFA, monounsaturated fatty acids; PUFA; polyunsaturated fatty acids; SFA, saturated fatty acids.

It has not escaped our notice that the membrane fluidity decrease we observed cannot be directly explained on the ground of these alterations in fatty acid composition. Schengrund et al. [64] recently reported a decrease in cholesterol and a related increase in GM1 ganglioside in erythrocyte membranes from ASD children, which could affect membrane fluidity. However, we failed to observe any change in membrane sialic acid - a component of GM1 ganglioside, in Au patients.

Na^+^/K^+^-ATPase maintains intracellular gradients of ions that are essential for cellular activities. Despite the crucial role of NKA in cellular metabolism and the fact that it accounts for approximately 30% of the total body energy consumption and for 50% brain energy consumption, very little is known about NKA in autism. In a mouse model of Angelman Syndrome, a neurodevelopmental disorder associated with autism, an intrinsic alteration of membrane properties of pyramidal neurons in hippocampal area CA1 has recently been observed [65]. Alterations were also observed in resting membrane potential, threshold potential, and action potential amplitude correlated with significant increases in the expression of the α1 subunit of Na^+^/K^+^-ATPase [64]. In postmortem tissues from different brain regions of autistic subjects, a specific increase in NKA in the frontal cortex and cerebellum was found. The authors suggested that such increase might be due to compensatory responses to increased intracellular calcium concentration in autism [66].

On the contrary, we showed a very significant reduction of erythrocyte NKA in Au compared to TD, in keeping with a similar report by Kurup and Kurup [67]. There is no overlap between the range values of the two groups, suggesting that this parameter might be a biomarker of autism. Future work should be addressed at understanding how sensitive and specific is the decrease of NKA as far as autism is concerned. A number of other factors may affect NKA; for example, a positive correlation between the molecular activity of Na^+^/K^+^-ATPase units and the membrane content of DHA has been shown [68] and a reduction of NKA has also been related to oxidative stress [69], [70]. Changes in ATPase activities might stem from sub-conformational changes in the enzymes depending on their microenvironment, indirectly reflecting changes in surrounding lipids and in membrane fluidity [71].

Noteworthy, some clinical features were correlated with some parameters of the lipidomic profile. In our study, hyperactivity is the clinical aspect found to be most highly related to erythrocyte membrane features. The higher the fluidity of the erythrocyte membrane and the lower the PUFA concentration, the greater was the hyperactivity level. Also, the severity of hyperactivity was directly and highly correlated with erythrocyte SFA and palmitic acid concentration. These data not only suggest that such disequilibrium in membrane fatty acid composition may be a useful tool to assess the severity of the autistic clinical picture, but also suggest possible therapeutic interventions with a tailored and balanced fatty acid intake. Two distinct double blind trials showed an improvement in hyperactivity score in autistic children treated with ω3 supplementation [72], [73]. Despite these encouraging results, a recent Cochrane meta-analysis stated that “to date there is no high quality evidence that omega-3 fatty acids supplementation is effective for improving core and associated symptoms of ASD” [74]. Nevertheless, our data clearly show an imbalance of membrane fatty acids and their correlation with relevant clinical features, thus pointing to the importance of restoring the membrane equilibrium. However, the intake of ω3 should be accompanied by antioxidant protection. For example, since our data also show the alteration of the redox balance of Au, supplementation of PUFA in the absence of antioxidant protection might paradoxically worsen the picture, as, in oxidative milieu, PUFA undergo a peroxidation process and may become, in turn, pro-oxidant. Also, omega-6/omega-3 balance might modulate neurotransmitters of the central nervous system: increased omega-3 fatty acid concentrations in cell membranes have been shown to affect serotonin and dopamine neurotransmission, especially in the prefrontal cortex [75]. Taking into account that serotoninergic and dopaminergic systems are deeply involved in ASD [76], [77], cell membrane lipid profile restoration could play a significant therapeutic role in improving some ASD features.

### Conclusions

Taken together, these results show significant erythrocyte membrane alterations in Au, at structural and functional levels, and an increase of lipid peroxidation markers. These alterations, and in particular the marked decrease in NKA, may play a role in the pathogenesis of ASD and potentially may be useful tools as peripheral biomarkers of ASD to be exploited for a more precise or an earlier diagnosis of ASD. Future work will be addressed at understanding the reason(s) for the impairment of the NKA and associated relevance to the pathogenesis of ASD. Finally, our data suggest the presence of systemic alterations in ASD, and emphasizes the possibility of an integrated approach aimed at correcting the membrane defects by means of nutraceutic tools.
